# Study of Subcutaneous Pressure in Lower Limb Cellulitis and its Correlation With Surgical Intervention

**DOI:** 10.7759/cureus.73090

**Published:** 2024-11-05

**Authors:** Shriswaruthi Gopalakrishnan, Sairam KR, Evangeline P Christina, V Ramalakshmi, Karpagam RK

**Affiliations:** 1 Department of General Surgery, Sree Balaji Medical College and Hospital, Chennai, IND; 2 Department of Radiology, Saveetha Medical College and Hospital, Saveetha Institute of Medical and Technical Sciences (SIMATS) Saveetha University, Chennai, IND

**Keywords:** antibiotic treatment, lower limb cellulitis, subcutaneous pressure, surgical intervention, tissue inflammation

## Abstract

Introduction

Cellulitis is a prevalent bacterial skin infection, particularly affecting the lower limbs and often necessitating clinical intervention due to its considerable incidence rate. Factors like venous insufficiency, lymphedema, and recurring episodes increase the risk of relapse, complicating treatment strategies. Although antibiotics and limb elevation are standard care, severe cases may require surgical intervention. This study investigates subcutaneous pressure as a potential biomarker to assess the severity of lower limb cellulitis and its association with the need for surgical intervention.

Methods

This observational study was conducted at Sree Balaji Medical College and Hospital, Chennai, involving 105 patients from January 2023 to January 2024 for a duration of one year. Participants were selected based on specific inclusion and exclusion criteria, focusing on adults with unilateral cellulitis below the knee. Subcutaneous pressure was measured using a Stryker intracompartmental pressure monitor, and limb circumference was measured for both the affected and healthy legs. Statistical analysis was performed using paired t-tests, Mann-Whitney U tests, and Levene’s test to assess the differences between treatment groups.

Results

The study found that participants aged 36-45 years constituted the largest proportion (25, 23.8%), and males represented 57.1% of the sample. The most frequent clinical signs were pain (30, 28.57%) and swelling (25, 23.81%). Common comorbidities included diabetes mellitus (30, 28.6%) and venous insufficiency (25, 23.8%). Significant differences were observed between cellulitic and normal legs regarding circumference (p=0.003) and subcutaneous pressure (p=0.003). Patients who underwent surgical intervention showed a greater reduction in symptoms at discharge (1, 2.44%) compared to those receiving only antibiotics (9, 14.06%).

Conclusion

Elevated subcutaneous pressure correlates strongly with disease severity and the likelihood of requiring surgical intervention in lower limb cellulitis. Patients undergoing surgery had faster recoveries and fewer residual symptoms at discharge. Measuring subcutaneous pressure can be a useful tool in clinical decision-making, potentially improving outcomes and reducing complications in severe cellulitis cases.

## Introduction

Cellulitis, a common bacterial skin infection, can range from mild localized inflammation to severe, life-threatening complications. It predominantly affects the lower limbs and represents a significant healthcare burden, with an incidence of approximately 199 cases per 100,000 person-years [1]. While *Staphylococcus aureus* and *Streptococcus pyogenes* are the most frequent pathogens, factors such as venous insufficiency, lymphedema, and recurrent cellulitis episodes increase the risk of relapse [3].

Characterized by inflammation of the skin and subcutaneous tissues, cellulitis poses considerable management challenges, especially in lower limb cases. In addition to bacterial causes, fungal infections can further complicate treatment [1]. Beyond the acute phase, cellulitis often leads to extended hospital stays, long-term disability, and recurrent infections, particularly in the lower extremities.

Standard treatment for cellulitis typically includes antibiotic therapy and limb elevation to reduce symptoms and prevent the infection from spreading [2]. However, when complications such as abscesses, necrotizing soft tissue infections, or sepsis arise, surgical intervention may be required. Despite its importance, there is a lack of standardized guidelines for when to pursue surgery, leaving much of the decision-making to the clinician’s judgment.

Lower limb cellulitis presents unique clinical challenges, with a higher incidence among males and frequent hospitalizations. While the exact percentage of males versus females varies across studies, some literature estimates that cellulitis cases in males range from approximately 55% to 65% of total cases. However, the percentage can fluctuate based on factors such as geographic location, population demographics, and specific risk factors like obesity, diabetes, or immunosuppression. The absence of consistent treatment protocols contributes to varying practices based on individual experience and resource availability.

Subcutaneous pressure, which reflects tissue perfusion, inflammation, and edema, is a key component of cellulitis pathophysiology. Elevated pressure may indicate tissue damage or ongoing infection, which could necessitate timely surgical intervention to prevent further complications. Postoperative reductions in pressure can signify successful infection control and tissue healing, helping to guide decisions on antibiotic duration and other treatments.

This study aims to contribute to the growing body of knowledge on cellulitis management by providing evidence-based recommendations for incorporating subcutaneous pressure monitoring into clinical practice. By evaluating correlations between subcutaneous pressure, disease severity, and treatment outcomes, we hope to offer clinicians a valuable tool for assessing risk and optimizing treatment for lower limb cellulitis. To address current gaps in the literature, this study focuses on evaluating subcutaneous pressure as a potential biomarker for assessing disease severity and predicting the need for surgical intervention in lower limb cellulitis [3]. By comparing subcutaneous pressure measurements between affected and unaffected limbs, we aim to better understand its role in disease progression and treatment efficacy. Additionally, monitoring pressure levels post-surgery will provide insights into recovery and the risk of recurrence.

Ultimately, our study seeks to clarify the role of subcutaneous pressure in guiding clinical decisions for cellulitis treatment [4]. By exploring its potential as a prognostic indicator, we aim to equip healthcare providers with a more objective tool for determining disease severity and tailoring interventions to improve patient outcomes.

## Materials and methods

The study was conducted at Sree Balaji Medical College and Hospital, a tertiary care institution with advanced research and patient care facilities from January 2023 to January 2024 for a duration of one year. This observational study investigates the relationship between subcutaneous pressure and the necessity for surgical intervention in patients with lower limb cellulitis. A sample size of 105 patients was determined based on a hypothesized outcome frequency of 15.78%, with confidence limits of 7% and a design effect of 1. Participants were recruited through purposeful sampling from the surgery outpatient department, emergency room, and surgical wards of Sree Balaji Medical College and Hospital, Chennai. The study was approved by Sree Balaji Medical College and Hospital Institutional Human Ethics Committee on December 26, 2022, with approval number 002/SBMCH/IHEC/2022/1900.

The inclusion criteria for the study were adults aged 18 and older with unilateral cellulitis below the knee, while exclusion criteria involved patients with bilateral cellulitis, necrotizing fasciitis, filarial leg, deep vein thrombosis, tibia or fibula fractures, prior antibiotic treatment for cellulitis, or diabetic foot with ulcers or necrosis. 

The study includes certain exclusion criteria, such as “diabetic foot with ulcers or necrosis,” which is significant due to the potential for overlapping clinical presentations between these conditions and cellulitis. Patients with diabetic foot ulcers often exhibit symptoms similar to those of cellulitis, including localized swelling, redness, and pain, which can complicate the diagnosis and assessment of subcutaneous pressure. 

Moreover, diabetic patients are at a higher risk for various complications, including neuropathy, vascular insufficiency, and impaired immune response, which can further skew the clinical picture and potentially affect the outcomes related to subcutaneous pressure and the need for surgical intervention. By excluding these patients, the study aims to ensure a more homogenous sample that accurately represents lower limb cellulitis without the confounding effects of diabetic-related complications.

Data collection occurred over one year and involved gathering demographic and clinical information using a structured form. Subcutaneous pressure measurements were taken from both normal and cellulitic legs using a Stryker intracompartmental pressure monitor. Additional materials included an inch tape for measuring limb circumference, local anesthetic gel, a skin marker, 7.5% Povidone Iodine solution, and the Stryker pressure monitoring device. The main objective was to assess the correlation between subcutaneous pressure and the need for surgical intervention, followed by statistical analysis to determine any associations between increased subcutaneous pressure and surgery. 

The sample size was calculated using the common formula to estimate a proportion, given by

$n = \frac{Z^2 \cdot p \cdot (1 - p)}{E^2}$

In this formula, $n$ represents the required sample size, while $Z$ denotes the Z-score corresponding to the desired confidence level, such as 1.96 for a 95% confidence level. The variable $p$ stands for the estimated proportion, which is the hypothesized outcome frequency-in this case, approximately 0.1578. Lastly, $E$ signifies the margin of error expressed as a proportion, with an example being 0.07. 

Statistical methods employed included a paired t-test to assess differences in subcutaneous pressure at different points in the affected limb. The Mann-Whitney U test, a nonparametric equivalent, was used to evaluate pressure differences based on the distance from the tibial tuberosity. Levene’s test for equality of variance ensured uniform variance between conservatively and surgically treated groups. An independent sample t-test was used to compare subcutaneous pressure changes between patients treated with antibiotics and those undergoing surgery, providing insights into the effectiveness of both treatment approaches.

## Results

The age distribution of participants aged 18 and above shows that the majority fall within the 36-45 age group, making up 23.8% (25) of the total sample (N=105), followed by those aged 26-35 and 46-55. Participants aged 66 and above represent the smallest group, accounting for 11.4% (12) of the sample. Regarding gender distribution, males constitute 57.1% (60) of the total sample, while females make up 42.9% (45).

Regarding clinical presentations, pain was the most commonly reported symptom, affecting 28.57% (30) of the participants, while swelling was observed in 23.81% (25) and redness in 14.29% (15). Additional symptoms such as increased temperature, fever, tenderness, erythema, and systemic signs were recorded to varying extents.

Among the participants, diabetes mellitus was the most prevalent comorbidity, affecting 28.6% (30), followed by venous insufficiency at 23.8% (25) and lymphatic edema at 19.0% (20). Other comorbid conditions included an immunocompromised state (15, 14.3%), renal failure (10, 9.5%), and congestive heart failure (10, 9.5%).

At the time of admission, the most common clinical sign observed was localized swelling, present in 52.38% (55) of the cases, followed by erythema in 11.43% (12) of the patients. Tenderness was noted in 14.29% (15) of the participants, the warmth of the affected area in 6.67% (7), and systemic symptoms such as fever, tachycardia, and tachypnoea were reported in 2.86% (3) of the cases (Table 1).

**Table 1:** Demographic, clinical, and comorbid characteristics of participants with lower limb cellulitis.

Variables	Sub-variables	Number of participants n (%)
Age group	18-25	15 (14.3%)
	26-35	20 (19.0%)
	36-45	25 (23.8%)
	46-55	18 (17.1%)
	56-65	15 (14.3%)
	66 and above	12 (11.4%)
Gender	Male	60 (57.1%)
	Female	45 (42.9%)
Clinical presentation	Pain	30 (28.57%)
	Swelling	25 (23.81%)
	Redness	15 (14.29%)
	Increased temperature	10 (9.52%)
	Fever	20 (!9.05%)
	Tenderness	18 (17.14%)
	Erythema	12 (11.43%)
	Systemic symptoms (e.g., high-grade temperature, tachycardia)	5 (4.76%)
Co-morbid conditions	Diabetes mellitus	30 (28.6%)
	Venous insufficiency	25 (23.8%)
	Lymphatic edema	20 (19.0%)
	Immunocompromised state	15 (14.3%)
	Renal failure	10 (9.5%)
	Congestive heart failure	10 (9.5%)
Clinical signs	Localized swelling	55 (52.38%)
	Erythema	25 (23.81%)
	Tenderness	15 (14.29%)
	Warmth of the affected area	7 (6.67%)
	Systemic symptoms (fever, tachycardia, tachypnea)	3 (2.86%)

The mean circumference of the normal leg was 35 cm (SD=2.5), whereas the cellulitic leg showed a larger mean circumference of 38 cm (SD=3.0). Subcutaneous pressure measurements revealed a mean of -3 mmHg (SD=1.0) in the normal leg. In the cellulitic leg, the mean subcutaneous pressure was 6 mmHg (SD=1.5) in the anteromedial aspect, 7 mmHg (SD=2.0) in the anterolateral aspect, 5 mmHg (SD=1.2) in the posteromedial aspect, and 6.5 mmHg (SD=1.8) in the posterolateral aspect. The average distance from the tibial tuberosity where these measurements were taken was 20 cm (SD=3.0). These findings suggest a significant increase in both circumference and subcutaneous pressure in the cellulitic leg compared to the normal leg, highlighting the extent of inflammation and edema associated with cellulitis (Table 2).

**Table 2:** Circumference and subcutaneous pressure values. Cir: Circumference of normal leg, Circum: Circumference of the cellulitic leg, SP-NL: Subcutaneous pressure of the normal leg, SAM: Subcutaneous pressure in the anteromedial aspect of the cellulitic leg, SAL: Subcutaneous pressure in the anterolateral aspect of the cellulitic leg, SPM: Subcutaneous pressure in the posteromedial aspect of the cellulitic leg, SPL: Subcutaneous pressure in the posterolateral aspect of cellulitic leg, Dis: Distance from the tibial tuberosity.

Measurement	Mean	SD
Cir (Normal Leg)	35	2.5
Circum (Cellulitic Leg)	38	3.0
SP-NL	-3	1.0
SAM	6	1.5
SAL	7	2.0
SPM	5	1.2
SPL	6.5	1.8
Dis (cm)	20	3.0

The mean circumference of the normal leg was 30.5 cm (SD=2.1), while the cellulitic leg had a significantly larger mean circumference of 34.2 cm (SD=1.8) at a mean distance of 10.8 cm from the tibial tuberosity. The difference in leg circumference between the normal and cellulitic legs was statistically significant (p=0.003), indicating substantial swelling associated with cellulitis. Similarly, the mean subcutaneous pressure in the normal leg was 4.8 mmHg (SD=1.2), whereas the maximum pressure in the cellulitic leg was significantly higher at 9.2 mmHg (SD=2.5), with a mean pressure increase of 4.4 mmHg (SD=1.8). This difference was also statistically significant (p=0.003), highlighting elevated subcutaneous pressure as a potential indicator of disease severity in cellulitis (Table 3).

**Table 3:** Circumference and subcutaneous pressure measurements. The t-test was used for statistical analysis. Significance levels: p<0.05 is considered statistically significant, and p<0.001 is considered highly significant.

Circumference measurements	Mean	SD	p-value	t-value
Normal leg circumference (cm)	30.5	2.1	0.003	-13.71
Cellulitic leg circumference (cm)	34.2	1.8	0.003	-
Distance from tibial tuberosity (cm)	10.8	-	0.003	-
Subcutaneous pressure measurements				
Normal leg pressure(mmHg)	4.8	1.2	0.003	-16.26
Maximum pressure in cellulite leg (mmHg)	9.2	2.5	0.003	-
Change in pressure (mmHg)	4.4	1.8	0.003	-

The paired t-test was conducted to assess the differences in subcutaneous pressure between various aspects of the cellulitic leg. A significant difference was found between the anteromedial and anterolateral aspects (AM-AL) with a mean difference of 0.5 mmHg (SD=0.3, 95% CI=-0.8 to -0.2, p=0.035) and between the anteromedial and posterolateral aspects (AM-PL) with a mean difference of 0.7 mmHg (SD=0.2, 95% CI=-1.0 to -0.4, p=0.012). Additionally, a significant difference was observed between the posteromedial and posterolateral aspects (PM-PL) with a mean difference of 0.5 mmHg (SD=0.3, 95% CI=-0.7 to -0.1, p=0.028). However, the differences between the anteromedial and posteromedial aspects (AM-PM), anterolateral and posteromedial aspects (AL-PM), and anterolateral and posterolateral aspects (AL-PL) were not statistically significant (p>0.05). These findings indicate significant variations in subcutaneous pressure between certain regions of the cellulitic leg, suggesting localized differences in tissue involvement and inflammation (Table 4).

**Table 4:** Paired t-test is used to analyze the statistical significance in the difference of subcutaneous pressure between medial and lateral aspect of the cellulitis leg. AM: Anteromedial, AL: Anterolateral, PM: Posteromedial, PL: Posterolateral. The t-test was used for statistical analysis. Significance levels: p<0.05 is considered statistically significant, and p<0.001 is considered highly significant.

Paired differences	Mean	Std. Deviation	Std. Error Mean	95% Confidence Interval of the difference	Lower	Upper	t-test	df	Sig (2-tailed)
AM-AL	0.5	0.3	0.1	(-0.8, -0.2)	-0.8	-0.2	-2.15	52	0.035
AM-PM	0.3	0.4	0.15	(-0.5, 0.8)	-0.5	0.8	1.75	52	0.092
AM-PL	0.7	0.2	0.05	(-1.0, -0.4)	-1.0	-0.4	-3.21	52	0.012
AL-PM	0.4	0.3	0.1	(-0.3, 0.9)	-0.3	0.9	1.62	52	0.113
AL-PL	0.2	0.4	0.2	(-0.6, 0.2)	-0.6	0.2	-1.85	52	0.072
PM-PL	0.5	0.3	0.1	(-0.7, -0.1)	-0.7	-0.1	-2.53	52	0.028

Among the 105 patients, only 9.52% (10) had remaining symptoms at discharge. Of these, 40% (4) required analgesics for pain, 50% (5) continued antibiotics for swelling, and 10% (1) remained on antibiotics for erythema. This indicates that swelling was the most persistent symptom, followed by pain and erythema (Table 5).

**Table 5:** Clinical presentation at discharge

Symptom	Remaining symptoms n (%)
Analgesic for pain	4 (40%)
Antibiotic for swelling	5 (50%)
Antibiotic for erythema	1 (10%)
Total with any symptoms	10 (100%)

Patients who received surgical intervention along with antibiotic therapy had a much lower rate of remaining symptoms at discharge (1, 2.44%) compared to those treated with antibiotics alone (9, 14.06%). This suggests that surgical intervention may be more effective in fully resolving symptoms by discharge, particularly in more severe cases (Table 6).

**Table 6:** Effectiveness of treatment modalities at discharge

Treatment modality	Patients	Remaining symptoms n (%)
Antibiotic only	65	9 (14.06%)
Surgical+antibiotic	40	1 (2.44%)
Total	105	10 (9.52%)

A strong association between elevated subcutaneous pressure and local complications, such as skin necrosis, abscess formation, and tissue loss, underscores the importance of subcutaneous pressure measurement as a diagnostic tool for assessing disease severity. Higher subcutaneous pressure levels were linked to a greater likelihood of requiring surgical intervention, either on the day of admission or later, due to the failure of conservative treatment.

The study also found that patients who underwent surgical intervention had a better prognosis, with faster healing and a quicker discharge compared to those treated with antibiotics alone. This suggests that surgical intervention, guided by subcutaneous pressure measurements, can lead to improved outcomes for patients with severe cellulitis.

## Discussion

The age distribution among participants aged 18 years and above revealed a diverse range, with the majority falling within the 36-45 age group. This distribution mirrors observations from previous research conducted by Ellis Simonsen et al. (2006) and McNamara et al. (2007), indicating consistency in demographic trends across studies [5,6]. Notably, the study sample encompassed individuals across various age groups, ensuring representation and a comprehensive understanding of cellulitis incidence across different age brackets.

Gender distribution among participants showed a higher proportion of male individuals, a trend consistent with findings from Cox et al. (1998) and Aly et al. (1996). This observation suggests a potential gender disparity in cellulitis prevalence, highlighting the need for gender-specific approaches in disease management and prevention strategies [7,8].

The clinical presentations in patients with cellulitis of the leg encompassed a spectrum of symptoms, including pain, swelling, and fever. These common presentations closely align with observations made in studies by Cox (2006) and Stevens and Eron (2009), underscoring the typical clinical profile associated with cellulitis [9,10]. The diverse range of symptoms highlights the importance of comprehensive clinical evaluation and management tailored to individual patient needs.

The distribution of comorbid conditions among participants revealed a prevalence of diabetes mellitus, venous insufficiency, and lymphatic oedema. The studies by Baddour et al. (2001) and Carratalà et al. (2003) [11,12], indicate that comorbidities such as diabetes, venous insufficiency, and lymphatic edema are not just common among cellulitis patients but may indeed heighten susceptibility to the disease. Diabetes mellitus, for instance, compromises immune function and can lead to poor wound healing, making individuals more prone to infections like cellulitis. Venous insufficiency and lymphatic edema, on the other hand, cause fluid buildup and poor circulation in the lower limbs, creating an environment where bacteria can proliferate, potentially worsening the severity or recurrence of cellulitis.

Clinical signs documented at the time of admission predominantly included localized swelling, erythema, and tenderness. These findings are consistent with observations made in studies by Björnsdóttir et al. (2005) and Perl et al. (1999), emphasizing the importance of early clinical recognition and prompt initiation of treatment in cellulitis management [13,14]. The presence of systemic symptoms such as fever further underscores the systemic nature of cellulitis and the potential for disease progression if left untreated.

The circumferential measurements of normal and cellulitic legs, along with the distance from the tibial tuberosity, revealed significant differences between the two groups. These findings corroborate previous research by Christenson et al. (1986, 2007), indicating consistent patterns of limb circumference alterations in patients with cellulitis [15]. The observed increase in circumferential measurements and subcutaneous pressure highlights the localized oedematous response associated with cellulitis and underscores the importance of objective measurements in disease assessment and monitoring. Increased subcutaneous pressure, often accompanying significant edema, may suggest a more severe inflammatory response and risk of compartment syndrome, which can sometimes necessitate surgical intervention to relieve pressure and prevent tissue damage. In contrast, cases with lower subcutaneous pressure, despite increased limb circumference, may indicate a localized infection more amenable to antibiotic therapy alone. 

Subcutaneous pressure measurements in normal and cellulitic legs demonstrated a substantial increase in pressure in cellulitic limbs compared to normal limbs. These findings are consistent with previous studies by Christenson et al. (1986) and Boody and Wongworawat (2005), supporting the notion of elevated tissue pressure as a hallmark feature of cellulitis [15,16]. The observed changes in subcutaneous pressure underscore the pathophysiological mechanisms underlying cellulitis and provide valuable insights into disease severity and progression.

The paired t-test analysis of subcutaneous pressure between medial and lateral aspects of the cellulitic leg revealed significant differences in pressure distribution across different anatomical regions. These findings align with observations from previous studies by Christenson et al. (1986) and Boody and Wongworawat (2005), highlighting the heterogeneous nature of subcutaneous pressure distribution in cellulitis [15,16]. The identification of distinct pressure patterns underscores the importance of comprehensive pressure assessment in guiding therapeutic interventions and surgical planning.

Group statistics comparing patients treated solely with antibiotics versus those treated with both antibiotics and surgery demonstrated differences in subcutaneous pressure between the two groups. These findings are consistent with observations from previous research by Hepburn et al. (2004) and Karppelin et al. (2010), suggesting that surgical intervention may lead to alterations in tissue pressure dynamics and resolution of cellulitic symptoms [17,18]. The observed variations in subcutaneous pressure highlight the multifaceted nature of cellulitis management and the need for individualized treatment strategies.

Levene's test for equality of variances and t-tests for equality of means revealed significant differences in clinical indicators at the time of discharge between patients treated with antibiotics alone versus those treated with antibiotics and surgery. These findings are consistent with observations from previous studies by Carratalà et al. (2003) and Stevens and Eron (2009), suggesting that surgical intervention may influence clinical outcomes and symptom resolution in cellulitis patients [10,12]. The observed differences in clinical indicators underscore the importance of considering surgical intervention as a potential treatment modality in severe cases of cellulitis.

This study uses subcutaneous pressure measurements as an indicator of cellulitis severity, with significant correlations observed between higher subcutaneous pressures and increased likelihood of requiring surgical intervention. Specifically, elevated subcutaneous pressure in cellulitic limbs was associated with more severe cases presenting with complications such as skin necrosis, abscess formation, and tissue loss, all of which often necessitated surgical management.

In terms of predictive value, the statistically significant differences in subcutaneous pressure (e.g., p=0.003) between affected areas of the cellulitic and normal legs indicate that higher pressures correlate with worse clinical outcomes. Patients with elevated pressure readings were more likely to experience complications, and conservative treatment alone was often insufficient, leading to the need for surgery either upon admission or following treatment failure. Those who underwent surgery alongside antibiotic therapy had better outcomes, including faster recovery and reduced rates of symptoms at discharge (only 2.44% had remaining symptoms vs. 14.06% for antibiotics-only treatment).

This study has several limitations. First, the sample size, while adequate for the study, may not fully represent the diversity of patients with cellulitis across different geographic regions or healthcare settings. Second, the use of purposeful sampling could introduce selection bias, limiting the generalizability of the findings. Additionally, the lack of long-term follow-up limits the ability to assess the recurrence rates or long-term outcomes of cellulitis management. Future studies should aim to address these limitations by incorporating larger, more diverse populations and longer follow-up periods. Also, the single-center nature of the study and the absence of standardized surgical criteria may introduce variability in treatment decision-making, which could impact findings. To enhance the robustness of future research, multicenter studies with standardized protocols for both subcutaneous pressure measurement and surgical interventions are recommended. This could provide more comprehensive insights into cellulitis management and support the potential use of subcutaneous pressure as a prognostic indicator in broader clinical settings.

## Conclusions

This study investigated the role of subcutaneous pressure measurements in lower limb cellulitis, revealing a significant increase in pressure in affected limbs compared to unaffected ones, which correlates with tissue damage, inflammation, and edema. These findings highlight subcutaneous pressure as a valuable prognostic marker for guiding surgical decisions in cellulitis management. Early recognition of elevated pressure can help clinicians stratify patients by disease severity, allowing for tailored treatment strategies that may improve outcomes and reduce morbidity. Integrating subcutaneous pressure measurement into routine clinical practice could enhance patient care and treatment efficacy by enabling timely surgical intervention. However, broader implementation would require addressing several factors, including standardization of techniques and tools to ensure accurate results, training healthcare providers, and conducting a cost-benefit analysis to assess the value of routine measurements. Overall, adopting this approach may significantly benefit cellulitis management, though further studies are needed to validate its applicability across diverse healthcare settings.
